# On the Effects of Bloodletting on the Young Subject

**Published:** 1848-02

**Authors:** 


*On the effects of Bloodletting on the Young Subject.* By John B. Beck, M. D., Professor of Materia Medica and Medical Jurisprudence, in the College of Physicians and Surgeons of N. Y.

There is no subject, perhaps, so deeply interesting to the practical Physician, as the Effects of Bloodletting on the human system, and the various uses to which it may be applied in the management of disease. In promptness and power, it exceeds all other agents, and its capacity for doing good or harm is proportionally great. It is resorted to, also, at every period of life, and by some it is even prescribed with equal, if not more freedom in children than in adults. It becomes, then, a question of the greatest moment to determine, if possible, whether the age of the patient has any influence in modifying its effects. And this is the subject upon which I propose to make a few remarks.

That the youngest child can sustain the loss of blood within certain limits, as well as the adult, is manifest from a variety of facts. Thus children are sometimes born in a state of asphyxia from apoplexy. On dividing the cord and letting a moderate quantity of blood, respiration is established, and every thing does well. Again, not unfrequently from not applying the ligature sufficiently tight around the cord, or from the cord contracting and thus loosening the ligature, hæmorrhage takes place, and yet no injurious consequences result. Besides this, we know that in cases of disease, the youngest children may be bled, not merely without injury, but with advantage. When, however, the loss of blood is carried beyond these limits, important peculiarities are observed, showing a difference in the effects produced in the young subject, from those in the adult.

*The first peculiarity is, that the young subject does not bear the loss of considerable quantities of blood, so well as the adult.* I am not aware that children fall into a state of syncope from the loss of blood more readily than adults; but when syncope does come on, it is very certain that they do not recover from it so readily, and they are always in more or less danger. In the adult, syncope from the loss of blood, unless the quantity be very large, is a state which, as a general rule, is attended with little or no danger, and from which the patient speedily recovers. Hence it is that physicians are continually in the habit of inducing it in the management of certain forms of disease, and not merely with impunity, but with evident advantage. In the young subject it is not so, and it is a state always attended with hazard. If the child recover from it, it does so very slowly, and every now and then it sinks irretrievably under its influence. That this is a fact, is confirmed by abundant testimony, on the part of those who have taken the trouble to make observations. Dr. Marshall Hall, in speaking on this subject, says, "In infancy, the stateof syncope (from the loss of blood) is a state of danger."* Evansell and Maunsell remark, "As a general rule, it is well to stop the flow of blood when decided pallor takes place, without waiting for actual fainting, from which children do not quickly recover."† Armstrong says, "Do not bleed to actual syncope in children, as they are apt to fall into convulsions, of which they may die. Children do not recruit from very large bleedings like adults."‡ Dr. Ryan observes, "The abstraction of blood in cases of infants and children until fainting occurs, is the worst practice that can be imagined, as convulsions or death may be produced."§ Indeed, the general fact admits of no question ; and the reason is obvious enough, if we reflect for a moment upon the nature of the agent, and at the same time compare it with the susceptibility of the subject. Carried to the point of syncope, bloodletting is one of the most direct, speedy, and profound sedatives that we have in our possession. In a few moments, it reduces the subject from a state of perfect health or the high excitement of disease, to the state of temporary death. Now it is very evident that the capability of recovering from such a state, must be just in proportion to the powers of the constitution. From the very nature of its organization, therefore, it is obvious that the system of the child

---

* Researches on the Morbid and Curative Effects of the Lass of Blood, by M. Hall. M. D., p. 87.

† on the Management of Diseases of Children, p. 107.

‡ Lectures, &c., by John Armstrong, M. D., p. 387.

§ Manual of Midwifery, by M. Ryan, M. D., p. 475.

cannot sustain so well as the adult a shock so sudden and powerful as this.

*The second peculiarity attending the loss of blood in the young subject, is, that the nervous system is more powerfully affected than in the adult.* The evidence of this is, that convulsions and coma more frequently occur after the loss of blood in children, than in adults. In the adult, both of these occurrences sometimes take place, more especially convulsions. Thus, for example, puerperal hæmorrhage is not unfrequently followed by them. I have witnessed the same thing in a gentleman of irritable habit, who had been bled too largely from the arm. He had lost about a quart of blood, when incipient syncope came on, followed immediately by a violent convulsion. In children, however, these occurrences are much more common; and the reason, no doubt, is the greater predominance, as well as impressibility of the nervous system. A great variety of causes, we know, will induce convulsions in a child, and among these, exhaustion is a very common one. With regard to coma, too, this may be brought on in children by any debilitating cause. A striking illustration of this we see now and then in diarrhœa, which has been continued too long. In these cases, the brain becomes suddenly affected, and a state of stupor or coma is induced, which not unfrequently is mistaken for Hydrocephalus. The same thing occurs from the loss of too much blood.

*The third peculiarity is, that the repetition of bloodletting is not so well borne by the child as the adult.* A child of good constitution and ordinary strength, may bear a first bleeding, perhaps quite as well as an adult. Under particular circumstances, too, of disease, a second may be borne dery well. Beyond this, as a general rule, it will be found, I think, that the child cannot well sustain the loss of blood. On this point, I believe, there is little or no difference of opinion among men of judgment and observation. Dr. John Clarke says, "Very young children bear very well the loss of blood even to fainting, once or twice, but they ill bear a more frequent repetition of bleeding. Their powers sink under it, and by no art can it be replaced."* Marshall Hall says, "In infancy, a second or third bloodletting is borne with difficulty."† Evanson and Maunsell say, "Repetitions of bloodletting are not well borne by the child."‡

*The fourth peculiarity is, that the effects of local bloodletting, especially leeching, are different upon the child, from what they are upon the adult.* In the adult the effect of leeching is in a great measure local, and it is not usually resorted to until after general bloodletting is considered inadmissible. In a child, on the contrary, it produces very much the same effect as a general bleeding. From the greater vascularity of the skin, too, the amount of blood lost by a leech, applied to a young subject, is much greater than in the adult,

---

* Commentaries on the Diseases of Children, p. 103.

† Researches on the Loss of Blood, p. 87.

‡ On the Diseases of Children, p. 108.

and it is frequently much more difficult to arrest the hæmorrhage from it. The general effect, then, of leeching, on the young subject, is much greater than upon the adult. Hence it is that cases are so frequently, in which children die from leeching. Of this we have numerous cases on record. Dr. Christison says, "I have twice known children bled to death in Hospital practice, the nurse having labored under a common prejudice among their craft, that leech-bites cannot bleed to much."* Pereira states, that "in two cases of infants, I have seen exhaustion with insufficient reaction, consequent on hæmorrhage after a leech-bite, terminate fatally."† Ryan says, "The loss of blood from a single leech-bite has caused the death of a child."‡

From the foregoing, then, it would seem, that although a child may bear the loss of certain quantities of blood, perhaps quite as well as the adult, when carried beyond this, they do not bear it so well, nor do they bear the repeated and continued loss of blood so well; and under these circumstances, dangerous and even fatal consequences are apt to ensue. In other words, bloodletting is an agent which operates with more power, and is attended with more danger in the child than in the adult.

If all this be so, then some conclusions may be drawn with regard to the practical application of this agent, which, to the young practitioner at least, may be of some importance.

1. Great caution should be exercised in bleeding children to the point of syncope. If the state of syncope be attended with the danger already alluded to, it is very certain that nothing can justify us in producing it, unless it be determined that it is essential to the management and cure of the case. Now, that in most cases, even of decided inflammation, it is not necessary to carry bloodletting to this extent, is very certain. We know that it is not so in the adult, and it evidently cannot be so in the child. As a general rule therefore, it cannot be required. By some high authorities, however, it is supposed that under certain conditions of diseased action, the safety of the patient depends upon the production of syncope. Thus, for example, in croup, bleeding ad deliquium has been insisted upon by the late Dr, Bayley of New-York,§ Dr. Dick of Alexandria,|| and Dr, Ferriar of Manchester. The latter especially speaks of it, as "the essential point of the cure, without which no relief can be effected."¶ If in any disease the practice be justifiable, it certainly is in this, and it cannot be denied, that in a great number of instances, it has been resorted to with safety. Notwithstanding this, general experience has abundantly established the

* Dispensatory, p. 492.
† Materia Medica, Vol. II., p. 769.
‡ Manual of Midwifery, p. 475.
§ New York Medical Repository, vol. 12, p. 331.
|| Barton's Med. and Phys. Jour,
¶ Medical Histories and Reflections, by John Ferriar, M. D., p. 371. American Edition.

fact, that even here it is not necessary, and that all the beneficially sedative effects of the remedy may be obtained, without going to this extent. On this point there appears to be, at the present time a pretty general concurrence of opinion among enlightened practitioners, and the rule of practice ought to be, *never* in any case to bleed to syncope, but to stop as soon as paleness of the lips and cheeks comes on. In this way, all the good of bloodletting is secured, while the risks of syncope are avoided.

2. To determine the precise amount of blood proper to be drawn is a matter of much greater nicety, and involves more serious consequences in the child, than in the adult. In the adult, the loss of a little more blood than is necessary, as a general rule, is a matter of no very great consequence. In the child on the contrary, it may prove fatal. In the adult, too, we have means of judging how far it ought to be carried, which we have not in the child. Thus, for example, the pulse, which in the adult is so valuable a guide in these cases, cannot be depended upon at all in the child. It is always, therefore, a very nice and difficult problem in practical medicine, how to adjust properly in a child the amount of blood necessary to be drawn, to the exact wants of the case. Now there are only two ways in which this can be done. The first is, by fixing upon a certain amount as suitable to different ages. The second is, to judge by the actual effects produced at the time of taking the blood.

With regard to the first of these modes, it is evident that it must be a very unsatisfactory guide, if we recollect that no two constitutions are precisely alike, and that there is every difference in the capacity of different systems, even in the same disease, to bear the loss of blood. Then, again, the same disease exists in different degrees of violence, and of course requirs a modification in the amount of depletion. Besides all this, different diseases do not require and cannot tolerate the same loss of blood. A general standard, then, founded upon the age of the patient, is really good for nothing, except as a mere approximation. In individual cases it must be inapplicable. Hence it is, that all those standards laid down by authors differ so much from one another, and must necessarily do so. If blood be taken by leeches, the difficulty is still further increased, from the circumstance that the desired quantity can hardly ever be obtained with any degree of precision: if it is so, it is purely by accident. That this must be so is evident, if we recollect the variable quntaties of blood drawn by the leeches themselves, and more especially the greater differences in the after-bleedings.

It is not yet settled, I believe, exactly how much blood a leech will draw. Christison says, "Twice as much blood may be usually drawn by fomentations, as by the suction of the leech. A single leech, when applied successfully, may thus be held to draw, from first to last, *about half an ounce of blood on an average*."* According to Evanson and Maunsell, "the quantity of blood obtained by a good leech, allowed to bleed for half an hour, may be estimated *at one ounce*."† Mr. Pereira says, "I believe *four drachms* to be the maximum. On an average, I do not think we ought to estimate it at more than *a drachm and a half*;"‡ i. e., the quantity taken by the leech itself, without reference to the after-bleeding. Now the fact is, it is impossible to specify the amount of blood drawn, either by the leech itself or in consequence of the subsequent bleedings. Leeches differ in their size very greatly, and there must, of course, be a great difference in the quantity of blood they are capable of taking. Then, again, there is every difference in the after-bleedings, depending on the vascularity of the skin, the part of the body to which they are applied, and various other circumstances. From all this, it is evident how unsafe it must be to draw blood from a child, according to any average standard.

With regard to the second mode, that of judging of the extent to which it should be carried by the effects produced at the time : in many cases this answers exceedingly well. In inflammatory complaints, where the full effect of the loss of blood may be necessary, the rule can be satisfactorily applied, and the best plan is to bleed in the erect posture, until pallor of the face comes on, without producing actual syncope. In the adult, according to Marshall Hall, the production of actual syncope constitutes the criterion as to the exact amount which the case requires, as well as of the capacity of the system to bear the loss of blood, and therefore he recommends this as the rule for the due administration of the remedy. Now, that this will not answer, must be obvious to every one. Every practitioner knows that cases are continually occurring, in which actual syncope comes on after the loss of a few ounces of blood, when large quantities are afterwards required to be drawn. In children, of course, the rule cannot be applicable. In them, the loss of so much blood as to bring on only approaching syncope might not only be unnecessary, but be attended with danger. From all this, then, it would appear that we are not in possession of any precise mode of determining how much blood ought in all cases to be taken in children ; and this shows the necessity of great caution and the exercise of sound judgment, in the use of the remedy.

3. From the uncertainty in estimating the quantity of blood lost by leeches, and the dangers attending the loss of two much from them in children, too great caution cannot be exercised in their use. From the manner in which leeches are ordered by some physicians in the diseases of children, one would be led to suppose that no harm could ever result from them. From the ease, too, with which they may be prescribed, and the appearance ofenergy which

* Dispensatory, p. 462.

† Practical Treatise on on Children, &c., p. 106.

‡ Materia Medica, vol. 2. p. 769.

it gives to the practitioner, it is be feared that not unfrequently they are used without being actually necessary, and even when necessary, they are suffered to draw blood without sufficient regard to the quantity which may be be lost. Now it should always be recollected, as already stated, that leeches operate differently on the child from what they do on the adult. In the latter, they are in a great measure local in their action, and may be, and generally are used, when general bleedly is contra-indicated. In the child. on the contrary, they act in the same way as general bleeding. Their sedative effects, therefore, upon the constitution of the child, are much greater; and if suffered to bleed beyond a certain limit, they endanger life. On these accounts, it is more necessary to be cautious in the use of them in children, than in adults. It is not my intention to go into any particulars, in relation to the mode of conducting the process of leeching. There are a few points, however, of a practical character, connected wih this subject, which may not be unworthy of notice. 1. When leeches are applied to a child, the patient should always be placed in the erect posture. The same rule indeed should be observed, in whatever way blood is drawn. If it be a fact that leeches act like general bloodletting upon the child, the propriety of this rule must be obvious; and it is the more necessary to insist upon it, because it is hardly ever observed. As soon as any paleness of the lips or face appears, the child should be placed in the recumbent posture, and the bleeding arrested. 2. When leeches are applied to a child, the patient should never be left until after the flow of blood is completely stopped. 3. Leeches should never be applied at bed-time, and suffered to bleed during the night. In this way, the patient has, in more cases than one, bled to death. If applied late at night, they should be watched just as in the daytime. 4. As a general rule, leeches should not be applied to soft parts destitute of support from underneath, in consequence of the difficulty of making pressure sufficient to arrest the hæmorrhage. The importance of this was first noticed by Dr. Cheyne, who advises to be applied in croup, not to the neck itself, but over the clavicle, sternum, or ribs.* 5. Leeches sometimes open into arteries, and dangerous hæmorrhage has ensued from this cause. A case of this ktnd happened, in which the temporal artery was thus open, and Sir Astley Cooper was obliged to divide the artery before the hæmorrhage could be arrested.† In all cases, therefore, the progress of the bleeding should be carefully watched.

4. If bloodletting be so profound a sedative to children, it is evident that it is capable of doing a vast deal of harm in cases unsuited to its use, and that it requires a very nice discrimination of the character cf the case, before it can be used with safety. This may appear very commonplace; bat, commonplace at it is, it is to be

* Patholog of the Larynxy and Bronchia, by John Cheyne, M. D., p. 57.

† Johnson's Med. Chir. Rev., Vol. 9, p. 71.

feared that it is not sufficiently borne in mind in actual practice. The presence of inflammation or congestion is generally considered a condition justifying and requiring resort to bloodletting, and so indeed, as a general rule, it is; but it is not so universally. Thus, for example, the inflammation attending scarlatina does not usually require or bear well the loss of blood ; and there can be no question that, in this complaint, many a child has been sacrificed by a resort to this remedy. Then, again, symptoms analogous to those produced by inflammation or congestion results from a cause directly the opposite, viz : irritation or mere exhaustion, Illustrations of this we see frequently in affections of the head in children, convulsions, &c. In these cases, if the cause of the difficulty be mistaken and depletion be resorted to, the result may be fatal, All this shows that, before bloodletting is used in children, the nature of the case should be investigated more nicely even than in the adult.

5. In the use of bloodletting in the young subject, especial regard should be had to their constitutions, as well as their mode of living. No principle is better understood, or ought to be so, even in adults, than that in the use of debilitating remedies, due regard should be had to the powers of the system. No practice is safe which does not take into consideration the relative capacity of the system to bear them ; otherwise the remedies may be more fatal than the disease for which they are prescribed. Now we know that in the adult there is every difference in this respect. In the management of the same disease accordingly in different individuals, a very different course of treatment is necessary, if not in the remedies themselves, at least in the extent to which they are carried. Iu the young subject this is still more necessary. Children whose constitutions are naturally feeble and vicious, or have been enfeebled by debilitating causes, such as poor diet, confined air, &c., sink very readily undea the influence of depressing remedies. In these bloodletting is badly borne, and should never be resorted to unless absolutely necessary, and then in moderate quantities,

6. Great caution should be exercised in the repetition of bloodletting. After what has been already said in relation to the effects of repeated bloodletting on the young subject, I should not again allude to it, were it not notice the opinions of an eminent authority. Dr. Rush, in his "Defence of Bloodletting," makes the following statement : "I could mention many more instances in which bloodletting has snatched from the grave children under three or four months old, by being used three to five times in the ordinary course of their acute diseases."* That the children alluded to by Dr. Rush survived this treatment I do not doubt , but that these repeated bleedings were necessary, I can hardly believe. At any rate, a practice like this, if generally adopted, would, in my humble opinion, end in the most disastrous results.

* Med. Obs. and Inqs., vol. 4, p. 300.

In concluding this paper, I trust it may not be thought that I am opposed to the use of bloodletting in the diseases of children. The physician who discards this agent, understands but poorly his profession or the duty whioh he owes his patients. The proper use of a remedy, however, is one thing, the abuse of it is another; and I must express the opinion, founded on no small observation, that it is frequently resorted to in children when it is unnecessary—when necessary, it is often carried too far—and that in its general use, there is frequently an absence of precision and care, whieh in many cases renders it a most dangerous remedy. With regard to the use of bloodletting generally in this country, there can be no doubt that the authority of Dr. Rush has exerted an influence the most deleterious. That it should have done so is not surprising. Living at a time when medicine was yet in its infancy among us—at the head of the oldest and most influential of our medical schools and attracting by his enthusiasm and his eloquence a large proportion of the students of the country, his sway for a series of years was unlimited, and his sanguinary precepts and his still more sanguinary practice† were speedily diffused from one end of the country to the other. Although sad experience has long since exposed the fallacy, as well as danger of his doctrines, yet many of the evil consequences of them are still to be met with: and not the least of these, it appears to me, is the opportunity which they have, indirectly at least, afforded for the prevalence of quackery, It is a part of our

---

† To justify the language used above, and which may be considered too strong by some, let me make a quotation or two from Dr. Rush's celebrated "Defence of Bloodletttng." "Bleeding should be conti ued while the symptoms which first indicated it continue, should it be until four-fifths of the blood contained in the body are drawn away." Med. Obs. and Inq., vol. 4, p. 353 The amount of blood in an adult is estimated at about 32 lbs. Four-fifths is over 24 lbs!

Again, in enumerating the advantages of bloodletting, he says: "In cases where bleeding does not cure, it may be used with advantage as a *palliative* remedy. Many diseases induce death in a full and highly excited state of system. Here opinm does harm, while bleeding affords certain relief. It belongs to this remedy, in such cases, to save pain, to relieve convulsions, to compose the mind, to protract the use of reason, to induce sleep, and thus to smooth the passage out of life." Med. Obs. and Inqs., vol. 4, p. 357. In other words, if I understand him, ode of the advantages of bleeding is, that it makes persons die easily! This reminds me of a melancholy case which I once witnessed. A young gentleman, about eighteen years of age, had been suffering about three months under organic disease of the brain. During this peroid he had been subjected to every kind of treatment. Bloodletting, emetics, cathartics, mercurials' tonics, &c., had all been used in succession, but without arresting at all the progress of the disease, and he had now become stone blind, was paralytic, and reduced to the extremest state of emaciation and debility. In short he was barely kept alive by the use of stimulants. In this state of things a friendly doctor happened to drop in, and expressed the opinion that the disease was inflammation of the brain, and that a good bleeding would relieve him. Notwithstanding the urgent remonstrances of the attending physician, that the result would be almost immediate death, the idea took with his friends, and he was bled by the doctor who suggested the practice. As might have been expected, in about six hours he was a corpse, and the great consolation seemed to be that he died so easily! Verily, on becoming acquainted with such practice, one would be tempted to believe that the Emperor Nero must have been a very tender hearted man in condeming Seneca to so pleasant a mode of terminating his existence as bleeding to death. For the particulars see the Annals of Tacitus, Book 15, Sec. 60.

nature to from extreme to another. When an error is once exposed we are apt to go immediately to its opposite, inferring that what is the reverse of wrong must necessarily be right; and so it has been in regard to bloodletting. The public having been made acquainted with the evils of the practice of Dr. Rush, a prejudice, if not general, at least very extensive, has been created against the remedy itself, and empirics, always ready to play upon the weaknesses and prejudices of the community, have seized upon it for the mere purposes of traffic. Accordingly, the land is now filled with a set of men who pretend to practice medicine, without resorting not merely to bloodletting, but many of the other remedies sanctioned by long and tried experience. And what is melancholy, but true, they find a ready sympathy in a large portion of the community. Whether I am too severe in attributing the popular empiricism of the day to the influence of Dr. Rush, must be left to the judgement of the profession. One thing, however, is very certain, and which we see illustrated every day. Whenever a person has been overtaxed with active medicine, he is apt to discard all belief in medicine generally, and he is then ready to fall into any absurdity. It is with medicine as it is with religion. Superstition once thrown off, infidelity follows, and the result in both cases is the same. Calm reflection and rational inquiry are out of the question, and boasted independence speedily becomes the easy prey of the knave and the empiric.—*N. Y. Annalist.*

